# Understanding the medication safety challenges for patients with mental illness in primary care: a scoping review

**DOI:** 10.1186/s12888-023-04850-5

**Published:** 2023-06-12

**Authors:** Matthew J. Ayre, Penny J. Lewis, Richard N. Keers

**Affiliations:** 1 Division of Pharmacy and Optometry, School of Health Sciences, Faculty of Biology, Medicine and Health, The University of Manchester, Manchester, UK; 2 NIHR Greater Manchester Patient Safety Translational Research Centre, Academic Health Science Centre (MAHSC), The University of Manchester, Manchester, UK; 3 Manchester University NHS Foundation Trust, Manchester, UK; 4 Suicide, Risk and Safety Research Unit, Greater Manchester Mental Health NHS Foundation Trust, Manchester, UK

**Keywords:** Mental illness, Mental health, Psychiatry, Medication safety, Prescribing safety, Primary care, General practice, Community pharmacy, Community mental health team, Nursing home

## Abstract

**Background:**

Mental illness and medication safety are key priorities for healthcare systems around the world. Despite most patients with mental illness being treated exclusively in primary care, our understanding of medication safety challenges in this setting is fragmented.

**Method:**

Six electronic databases were searched between January 2000-January 2023. Google Scholar and reference lists of relevant/included studies were also screened for studies. Included studies reported data on epidemiology, aetiology, or interventions related to medication safety for patients with mental illness in primary care. Medication safety challenges were defined using the drug-related problems (DRPs) categorisation.

**Results:**

Seventy-nine studies were included with 77 (97.5%) reporting on epidemiology, 25 (31.6%) on aetiology, and 18 (22.8%) evaluated an intervention. Studies most commonly (33/79, 41.8%) originated from the United States of America (USA) with the most investigated DRP being non-adherence (62/79, 78.5%). General practice was the most common study setting (31/79, 39.2%) and patients with depression were a common focus (48/79, 60.8%). Aetiological data was presented as either causal (15/25, 60.0%) or as risk factors (10/25, 40.0%). Prescriber-related risk factors/causes were reported in 8/25 (32.0%) studies and patient-related risk factors/causes in 23/25 (92.0%) studies. Interventions to improve adherence rates (11/18, 61.1%) were the most evaluated. Specialist pharmacists provided the majority of interventions (10/18, 55.6%) with eight of these studies involving a medication review/monitoring service. All 18 interventions reported positive improvements on some medication safety outcomes but 6/18 reported little difference between groups for certain medication safety measures.

**Conclusion:**

Patients with mental illness are at risk of a variety of DRPs in primary care. However, to date, available research exploring DRPs has focused attention on non-adherence and potential prescribing safety issues in older patients with dementia. Our findings highlight the need for further research on the causes of preventable medication incidents and targeted interventions to improve medication safety for patients with mental illness in primary care.

**Supplementary Information:**

The online version contains supplementary material available at 10.1186/s12888-023-04850-5.

## Background

Medication safety is a healthcare priority across the globe with the World Health Organization (WHO) setting a global patient safety challenge with medication safety as a key theme [1]. The National Health Service (NHS) in the United Kingdom (UK) has also set a patient safety strategy with medication safety and mental health safety as two key areas for improvement [2]. The WHO have highlighted that the majority of deaths of people with serious mental illness (SMI) may be prevented with improvements in monitoring of physical health and medications, particularly their side effects [3].

It has been observed that patients with SMI such as schizophrenia and bipolar disorder have a considerable reduction in life expectancy of 10–20 years compared to the general population [4]. Patients with mental illness are more likely to die from comorbidities such as cardiovascular disease (CVD), infections, and suicide [5] with CVD being the leading cause of death in patients with SMI [6]. In addition to this, research has also shown that patients with mental illness are more likely to develop comorbidities such as diabetes [7]. Psychotropic medications are used first line to treat psychiatric illnesses [8] as they may help reduce symptoms, increase functioning, and prevent relapse of symptoms [9–11]. However, psychotropic medications carry unpleasant side effects such as antipsychotic related cardiovascular effects and metabolic syndromes [12] which contribute to the risk of developing comorbidities such as CVD, and increased mortality in this population [13]. Comorbidity can also increase the number of medicines being prescribed together which can lead to potentially dangerous interactions [14].

In recent years there has been increased attention in the published literature on medication safety for patients with mental illness in inpatient settings [15–20]. Unique risk factors associated with this patient group have been reported that can impact the safe use of medication including those related to patient-clinician relationships, patient behaviours, and impaired cognition [19, 21]. Use of psychotropic medication within mental health hospitals can result in harm [22], and it is known that these inpatients are subject to prescribing, monitoring, dispensing, and administration errors [19].

Primary care encompasses settings which *“practice in the context of family and community”* [23] and include services such as general practice, community pharmacy, community mental health teams (CMHT), and elderly care/nursing homes. Whilst several review papers have summarised the evidence concerning medication safety in mental health inpatient care [17, 24, 25], the available literature for primary care is fragmented despite the emergence of recent studies [26, 27]. This is important as 90% of patients with mental illness are treated solely in primary care [28], and this setting accounts for a greater estimated proportion of errors with medication in England per year than secondary care for the general population (38.4% compared to 19.9%) [29]. As many as four in ten patients from the general population come to harm in primary care and up to 80% of this is avoidable, with the most harmful errors around diagnosis and the prescribing/use of medication [30]. Within mental health populations, some psychotropic medications such as lithium require regular blood and physical health monitoring [31] however, only 40% of patients prescribed lithium in England receive the necessary health checks in primary care [32]. As general practitioners (GPs) may have a lack of formal training and knowledge regarding mental illnesses [21, 33] this may also contribute towards the emergence of medication safety challenges for this patient group. It is also unclear whether the same risk factors identified for medication safety challenges in secondary mental health care [19] may be applicable to primary care settings.

Therefore, the aim of this scoping review was to identify and describe the evidence base for the epidemiology, aetiology and evaluated remedial interventions addressing medication safety challenges that patients with mental illness experience within primary care, in order to identify future research targets.

## Methods

The scoping review methodology was guided by the five stage framework proposed by Arksey and O’Malley [34] and reported following the Preferred Reporting Items for Systematic Reviews and Meta-Analyses scoping review (PRISMA-ScR) extension checklist [35, 36].

### Research question

Preliminary searches of the literature were conducted, and the following three research questions were formulated:

1. What is the epidemiology of medication safety challenges for patients with mental illness in primary care?
2. What is the aetiology of these medication safety challenges for patients with mental illness in primary care?
3. What are the trialled interventions and their outcomes to improve medication safety for patients with mental illness in primary care?

### Identification of relevant studies

#### Databases

A search was conducted using six electronic databases: Embase, Medline, Cochrane reviews, PsycINFO, CINAHL, and Web of Science core collection. These databases provide good coverage of medico-scientific literature and the literature relating to health professionals [37, 38].

### Search strategy

The search terms were grouped into three main themes which were “mental health”, “primary care”, and “medication safety.” These themes were the foundations of the search strategy and variations of terminology were used with appropriate Boolean operators applied for example, “mental health OR mental illness.” Search dates were restricted to papers published from January 2000-January 2023 as the patient safety movement gathered momentum from the start of the new millennium [39–41]. An example of a search strategy used in one database can be found in Supplementary file 1. Additional papers were identified by screening reference lists of included and relevant studies (e.g. topical systematic reviews), as well as using the search engine, Google Scholar™. Only peer reviewed data was included in this review, so for this reason Grey literature was excluded [42]. A PRISMA flow diagram was used to report and map the numbers at each stage of the search and selection process [43].

### Definitions

The definitions of medication safety terms and primary care used for this scoping review can be seen in Table 1 below, with DRP being used as the overarching umbrella term to categorise safety challenges.

**Table 1:** Definitions of medication safety and primary care terms

Medication safety/primary care term	Definition
Adverse drug event*	An injury due to the use of a medication [44]
Adverse drug reaction*	A harmful/unpleasant reaction from the use of a medicinal product [45]
Drug-related problem (DRP)	Broad term which covers unnecessary medication, ineffective medication, additional drug therapy required, dose too high/low, non-adherence and adverse drug reactions. DRP is an umbrella term which encompasses both medication errors and adverse drug reactions [46]
Medication error	*“Any preventable event that may cause or lead to inappropriate medication use or patient harm while the medication is in the control of the health care professional, patient, or consumer. Such events may be related to professional practice, health care products, procedures, and systems, including prescribing, order communication, product labelling, packaging, and nomenclature, compounding, dispensing, distribution, administration, education, monitoring, and use”* [47]
Non-adherence	Any deviation (intentional or unintentional) from a prescribed medication regimen by a patient [48]
Potentially hazardous prescribing (PHP)	Prescribing events that have the potential to cause harm [49, 50]
Potentially inappropriate prescribing/medication (PIP/PIM)	Prescriptions/medication that *“introduces a significant risk of an adverse drug related event when there is evidence for an equally or more effective alternative medication”* [51]
Primary care	*“the provision of integrated, accessible health care services by clinicians who are accountable for addressing a large majority of personal health care needs, developing a sustained partnership with patients, and practicing in the context of family and community.”* [23]

*Only preventable incidents are of interest in this review

### Study selection

After the search strategy was applied to all six databases the returned results were exported into Endnote 20 software and the duplicates removed. The screening process was carried out by one reviewer (MJA) who manually screened all of the articles retrieved. Firstly, studies were excluded by title, secondly by abstract, and the remaining studies were carried forward for full-text review. Any studies that were ambiguous were separated and discussed with the research team as to whether they should be included. During the screening and review process all the papers were scrutinised against the following inclusion/exclusion criteria.

#### Studies were included that:

- were set in primary care i.e., general practice, community pharmacy, nursing/elderly care homes, ambulatory care, community services (e.g. CMHTs, community mental health clinics etc.)
- had a population with previously confirmed psychiatric diagnosis or confirmed diagnosis by formal screening
- reported on medication safety of psychotropic and/or non-psychotropic medication use in patients with a confirmed psychiatric diagnosis
- reported on at least one of the following: epidemiology and/or aetiology of medication safety challenges and/or evaluated interventions designed to improve medication safety
- presented epidemiological data as a rate (or data enabling a rate to be calculated)
- presented aetiological data qualitative/quantitatively such as causal or risk factor data
- presented interventions addressing one or more safety challenges with qualitative/quantitative medication safety outcomes reported
- were published between 1st January 2000 and 17th January 2023
- focused on medication safety challenges originating in primary care that were detected in secondary care (e.g. emergency department)
- reported on medication safety for one or more different medication classes (e.g. antipsychotics)

#### Studies were excluded that:

- were clinical case studies, clinical drug trials, opinion/commentary, book reviews, reviews (e.g. narrative, systematic)
- were based in a secondary care/inpatient context, outpatient hospital clinics and prisons
- included populations with a psychiatric diagnosis by proxy (e.g. confirmed depression via an antidepressant prescription)
- reported medication safety challenges during admission or on discharge from secondary care
- reported medication safety challenges in patients without mental illness or where data for patients with confirmed mental illness could not be extracted from the wider study population
- reported medication safety data on specific drug(s) alongside specific disease states in conjunction with mental illness (e.g. depression and antiretrovirals, depression and heart failure)
- presented only non-preventable adverse drug reactions/events or side effect profiles for medications (e.g. antipsychotic induced weight gain)
- focused on one drug (e.g. quetiapine)
- focused on one subtype of medication safety challenge (e.g. drug/dose omission errors)
- were non-English language studies

### Data extraction and charting

The data was extracted from the studies by one reviewer (MJA) using a standardised collection form as shown in Supplementary file 2. Extraction involved gathering general background information such as study title, country and primary care setting. The epidemiology and aetiology of errors, preventable harm caused, and interventions were also extracted. The majority of studies reported one medication safety outcome of non-adherence, so a random sample (20/54 studies) of these were evenly shared for independent extraction by RNK & PJL. The rest of the studies were all unique, so they were all taken forward for independent extraction. Any discrepancies in extraction were discussed as a team until a consensus was reached. This approach also supported the validation of key themes emerging from the dataset which guided the presentation of the results.

### Data analysis and summary

Extracted data was entered into a summary table presenting the key information: author & year, country, primary care setting(s), age of population, study population, relevant psychiatric diagnosis(es) in the study sample, medication safety challenges reported, and whether the study reported any epidemiological, aetiological, and/or remedial intervention medication safety data. Each represented country used predominantly similar terminology to describe primary healthcare settings seen in the UK; the terms internal medicine, internist, and ambulatory care required alignment with general practice as a comparable UK setting. Where participant ages were not readily available either the age in the dataset (mean acceptable) was used, or the age was reported as non-specified. Epidemiological and aetiological data was presented in the form of a narrative, with key figures of interest highlighted for DRP sub-types. The epidemiology narrative was presented according to DRP types such as non-adherence and PIP/PIM. Studies which evaluated an intervention only had baseline data reported in the epidemiological narrative. Interviews were included in epidemiology which followed a structured format and reported percentage rates. Aetiological data was derived from incident reports, interviews, and statistics from prevalence studies for DRP risk factors (correlations) and causal data. Risk factors were defined as data that presented quantitative correlations between measurable factor(s) and DRPs, and causal data was quantitative/qualitative data that provided underlying reasons for the emergence of DRPs. This was summarised into a table reporting safety challenges as either prescriber- or patient-related. Prescriber-related included any clinician orientated processes (e.g. prescribing) and patient-related included any domains and responsibilities of the patient (e.g. taking medication). Evaluated interventions were presented in a table outlining the country of origin, primary care setting, study population, aim of the intervention, and impact on any reported medication safety outcomes. Each master table for epidemiology, aetiology and intervention data was condensed into summary tables.

## Results

**Fig. 1:**
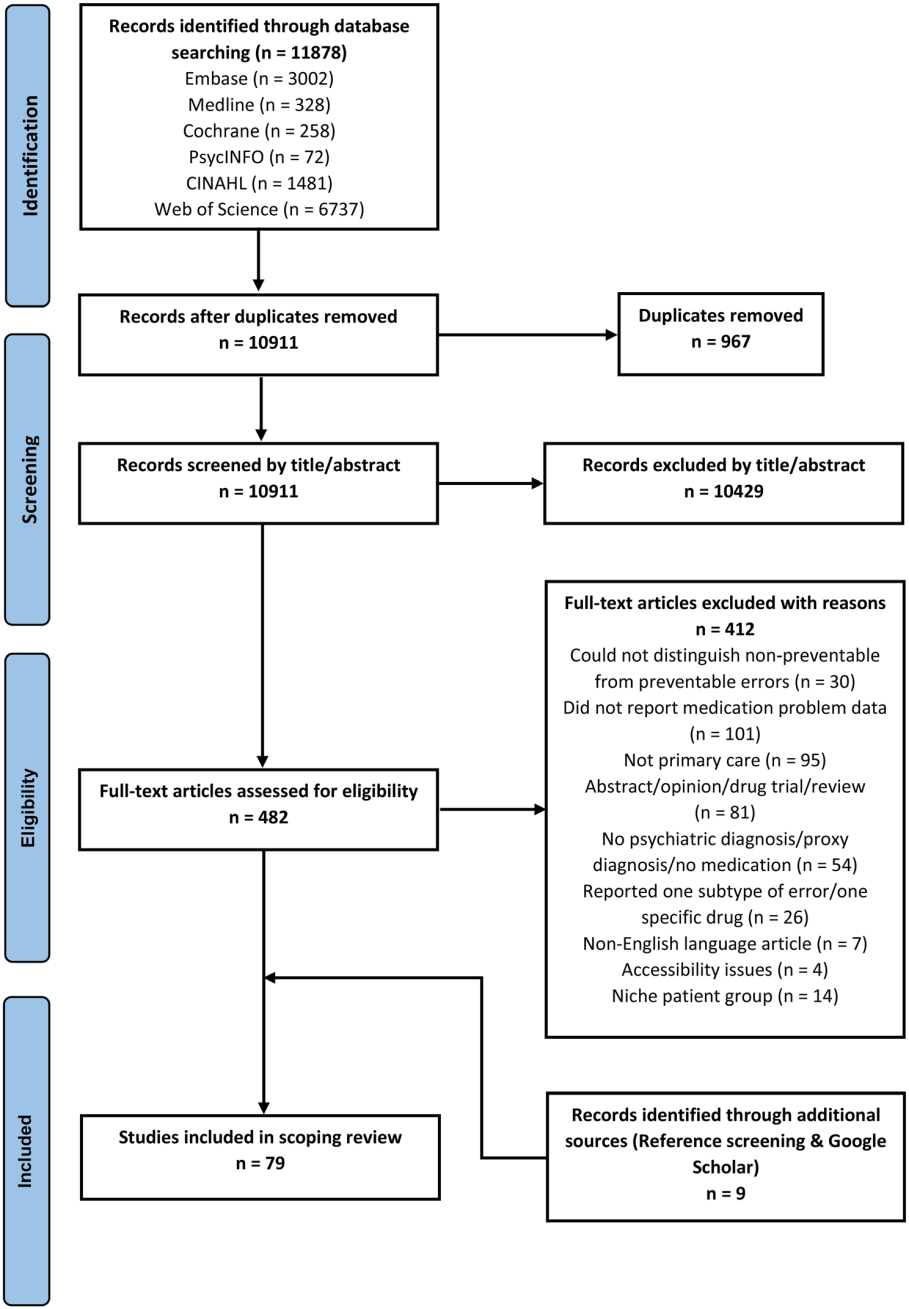
PRISMA flow diagram of identification, screening, and selection process Adapted [43]

A total of 11,878 articles were retrieved and, after duplicates were removed, 10,911 were screened by title and abstract. This resulted in 482 papers being taken forward for full-text review. During the full-text review stage, a total of 412 were excluded. Nine studies were identified through reference screening of relevant/included studies and searches in Google Scholar^™^. This resulted with a final number of 79 studies being included in the scoping review. Figure 1 provides a breakdown of the process, including reasons for exclusion at the full-text stage.

### Study characteristics

Study publication date distributions were 2000–2010 (21/79, 26.6%) and 2011 onwards (58/79, 73.4%). The included studies covered a wide geographical location as follows; USA (33/79, 41.8%) [52–84], UK (11/79, 13.9%) [26, 85–94], Australia (4/79, 5.1%) [95–98], Spain (4/79, 5.1%) [99–102], France (3/79, 3.8%) [103–105], Germany (3/79, 3.8%) [106–108], Netherlands (3/79, 3.8%) [109–111], Canada (2/79, 2.5%) [112, 113], India (2/79, 2.5%) [114, 115], Scotland (2/79, 2.5%) [116, 117], Slovenia (2/79, 2.5%) [118, 119], Sweden (1/79, 1.3%) [120], Belgium (1/79, 1.3%) [121], Brazil (1/79, 1.3%) [122], China (1/79, 1.3%) [123], Finland (1/79, 1.3%) [124], Israel (1/79, 1.3%) [125], New Zealand (1/79, 1.3%) [126], South Africa (1/79, 1.3%) [127], Taiwan (1/79, 1.3%) [128], and across eight European countries (1/79, 1.3%) [129]. The dataset summaries are presented in Supplementary file 3.

The three most commonly studied settings were general practice (31/79, 39.2%) followed by non-specific community-dwelling settings (22/79, 27.8%) and then community pharmacy and nursing homes (9/79, 11.4% each). Participant ages varied with the three most common being patients across the adult age range (at least 18+) (41/79, 51.9%), followed by older adults (aged 55+) (24/79, 30.4%) and then no age/no specific age (10/79, 12.7%) [26, 67, 75, 78, 83, 84, 88, 89, 93, 123].

The three most commonly studied populations were patients with mental illness (59/79, 74.7%), followed by elderly with comorbidities (one being a mental illness) (10/79, 12.7%) [52, 54, 56, 103–105, 124, 126, 128, 129] and then dementia with other comorbidities (5/79, 6.3%) [55, 86, 87, 97, 106]. Studies reported either one or more mental health diagnoses with 37 studies focusing on one and 42 reporting multiple diagnoses. Some studies focused specifically on patients with dementia (5/79) and others included dementia as an elderly comorbidity. The most commonly reported diagnoses were depression (48/79, 60.8%), followed by psychosis (23/79, 29.1%), anxiety disorders (21/79, 26.6%), bipolar disorder (20/79, 25.3%), dementia (15/79, 19.0%), and others such as personality disorders (8/79, 10.1%). Ten studies (12.7%) did not report/specify specific psychiatric diagnoses within their study population.

Out of the 79 included studies, 77 (97.5%) reported extractable epidemiological DRP data, 25/79 (31.6%) reported aetiological data, and 18/79 (22.8%) investigated and evaluated a remedial intervention. The most investigated DRP was non-adherence with 62/79 (78.5%) studies reporting data on this. The next most documented DRP was PIP/PIM/Potentially Hazardous Prescribing (PHP) with 20/79 studies (25.3%) reporting those. Four studies reported both non-adherence and PIP/PIM (5.1%) [95, 96, 106, 129]. A total of twelve studies (15.2%) reported on some other form of DRP such as a medication error (12/12, 100%), and a preventable adverse drug reaction/event (3/12, 25.0%).

### Epidemiology of drug-related problems

Seventy-seven studies reported quantitative epidemiological data for DRPs. A summary of the characteristics can be seen in Table 2. Some studies utilised multiple data collection methods (21/77, 27.3%) with the main methods as follows: medical/chart/pharmacy/prescription records (33/77, 42.9%), clinical/insurance databases (21/77, 27.3%), questionnaire/survey (19/77, 24.7%), interviews – providing % rates (17/77, 22.1%), patient medication reviews (6/77, 7.8%), technology e.g. electronic pill counter (3/77, 3.9%), and observation e.g. recording medication from home visits (1/77, 1.3%). The DRP investigated the most was non-adherence (60/77, 77.9%), followed by PIM (14/77, 18.1%), then medication errors (11/77, 14.3%), PIP (5/77, 6.5%), preventable adverse drug reactions/events (3/77, 3.9%), and PHP (1/77, 1.3%). A data summary of each epidemiological study can be viewed in Supplementary file 4.

**Table 2:** Characteristics summary of 77 epidemiology studies

Study Characteristic	Characteristic subcategory	No. of studies (%)
Year of Publication	2000–2010	21 (27.3)
	2011 onwards	56 (72.7)
Country of origin	USA	33 (42.9)
	UK	10 (13.0)
	Europe	21 (27.3)
	Other	13 (16.9)
Primary care setting	General Practice	31 (40.3)
	Community-dwelling	21 (27.3)
	Nursing Homes	9 (11.7)
	Community Pharmacy	8 (10.4)
	CMHT	4 (5.2)
	CMHC	3 (3.9)
	Ambulatory Care	1 (1.3)
Data collection method	Medical/Pharmacy/Prescription records	33 (42.9)
	Clinical/insurance databases	21 (27.3)
	Questionnaire/survey	19 (24.7)
	Interviews	17 (22.1)
	Medication reviews	6 (7.8)
	Technology	3 (3.9)
	Observation	1 (1.3)
DRP investigated	Non-adherence	60 (77.9)
	PIP/PIM/PHP	20 (26.0)
	ME	11 (14.3)
	Preventable ADR/ADE	3 (3.9)
Patient population	Patients with mental illness	58 (75.3)
	Elderly	10 (13.0)
	Dementia patients with comorbidities	5 (6.5)
	General population (mental health sub-population)	3 (3.9)
	Patients with chronic diseases	1 (1.3)
Psychiatric diagnoses within study population^#^	Depression	48 (62.3)
	Psychosis	23 (29.9)
	Anxiety disorders	21 (27.3)
	Bipolar disorder	20 (26.0)
	Dementia	14 (18.2)
	NS	9 (11.7)
	Others*	8 (10.4)

# Some studies reported multiple diagnoses; * e.g. ADHD/Personality disorders; ADE = Adverse drug event; ADR = Adverse drug reaction; CMHC = Community mental health clinic; CMHT = Community mental health team; DRP = Drug-related problem; ME = Medication error; NS = Nonspecified; PHP = Potentially hazardous prescribing; PIM = Potentially inappropriate medication; PIP = Potentially inappropriate prescribing; UK = United Kingdom; USA = United States of America

#### Non-adherence

Non-adherence was the most investigated medication safety challenge (60/77, 77.9%) with 53/60 (88.3%) of these studies focusing solely on non-adherence. A total of 56/60 (93.3%) non-adherence studies included patients with mental illness as the main population. The two diagnoses cited the most were depression (38/60, 63.3%), and then psychosis (20/60, 33.3%). The setting where the majority of non-adherence study data was exclusively collected were general practice settings (26/60, 43.3%) followed by non-specific community-dwelling settings (19/60, 31.7%), with no studies collecting data from multiple community settings.

Non-adherence rates were as low as 12.2% of patients out of a total of 49 participants [95] and as high as 97.8% of patients (45/46 participants) [122]. Miasso et al. also noted that non-adherence in Brazil to general psychotropics was 88.9% in primary health care [122]. Two studies reported similar initial antidepressant prescription collection rates of 85.0% in Spain [100] and 85.1% in Sweden [120], meaning similar non-adherence rates at the start of antidepressant treatment. Discontinuation of antidepressants over six months was reported to be half of patients with similar figures of 53% in Belgium [121] and 52.7% in Germany [108]. Antipsychotic non-adherence in the USA was reported to be as high as 71.0% [69] with Marcus et al. noting non-adherence to long-acting injectables to be 51.8% [64]. Sedative hypnotic non-adherence was found to be 6.3% in one study investigating adherence in patients with bipolar disorder in the USA [68]. Mood stabiliser non-adherence was reported in three USA studies, looking at a population with bipolar disorder, as being 19.3% [78], 24.1% [84], and as high as 82.3% [68]. Patients discontinuing stimulant medication in the USA was reported at a rate of 21.0% [81]. Of those (21.0%) discontinuing stimulant medication, many discontinued within one month of initiation (42.0%) and by six months 96.0% had discontinued [81].

#### Potentially inappropriate prescribing/medication

In total 20/77 (26.0%) studies reported PIP/PIM/PHP epidemiological data (14/20 PIM, 5/20 PIP, 1/20 PHP). Half of these studies reported the study populations as elderly with comorbidities (10/20, 50.0%). Five (5/20, 25.0%) studies focused specifically on patients with dementia [55, 86, 87, 97, 106] and 4/20 (20.0%) evaluated a broader range of psychiatric diagnoses [95, 96, 118, 119]. The final study used a clinical database with a mental health sub-population to investigate indicators of PHP [26]. The diagnosis cited the most was depression 14/20 (70.0%), followed by dementia 10/20 (50.0%). The main age of interest was elderly patients, with 15/20 (75.0%) investigating a population that were mostly aged 60 and above. Nursing homes were the most commonly reported study setting (8/20, 40.0%) [52, 55, 86, 97, 104, 105, 118, 124].

The percentage of antidepressants reported to be potentially inappropriate amongst elderly patients in one study across eight European countries (2005) was 20.0% [129], with one study reporting this to be as high as 43.7% in elderly patients [128]. PIP was seen with the use of first and second generation antipsychotics with studies reporting prevalence rates of 24.8% [97], 31.5% [104], and 53.4% [128] of antipsychotics being potentially inappropriate. One study specifically investigated potentially inappropriate neuroleptics and found 55.6% were inappropriate in patients with dementia, 37.5% in patients with diagnosed depression, and 10.2% in patients with mood disorders measured using a valid scale [105]. Inappropriate benzodiazepine and hypnotic prescribing were as high as 39.7% (17,977/45,242) and 48.4% (5,124/10,588) respectively, within an elderly population in Taiwan [128]. One study cited 50.0% of anxiolytic prescriptions being affected by PIP [104]. No studies reported any PIP/PIM data for mood stabilisers or stimulants.

#### Medication errors and adverse drug reactions/events

A total of 11/77 (14.3%) studies reported other DRP epidemiological data which were medication errors (11/11), preventable adverse drug reactions (2/11) and preventable adverse drug events (1/11) [26, 54, 55, 74, 85, 88, 95, 96, 106, 118, 124]. The two most frequently cited study settings were nursing homes (4/11, 36.4%) [54, 55, 118, 124] and general practice (4/11, 36.4%%) [26, 74, 88, 106]. Depression was the most cited psychiatric diagnosis (7/11, 63.6%). The study age of interest were commonly older patients with 5/11 (38.5%) reporting population ages of 65 and over [54, 55, 106, 118, 124], 3/11 (27.3%) studies examined an adult population [85, 95, 96], 2/11 (18.2%) studies did not specify a study population age range [26, 88], and one study (9.1%) examined a paediatric population ≤ 18 [74]. The study populations were patients with mental illness - including dementia specific (8/11, 72.7%) [55, 74, 85, 88, 95, 96, 106, 118], elderly with comorbidities (2/11, 18.2%) [54, 124], and 1/11 (9.1%) examined a general population clinical database [26]. The most cited medication error type was drug-drug interactions (7/11, 63.6%) [54, 55, 95, 96, 106, 118, 124], followed by dosage errors/dose too high/low (5/11, 45.5%) [54, 88, 95, 96, 106], then monitoring errors (4/11, 36.4%) [26, 74, 88, 96].

A study originating in Slovenia which included 24 patients found a total of 79 drug-drug interactions (18 major and 61 minor), with the highest number of major interactions per patient being five [118]. A study from Germany found 8% of patients with dementia were prescribed doses too high and 5% were prescribed doses too low [106]. Two of the four studies reporting monitoring issues reported additional tests being required for patients [88, 96]. Two out of 11 studies (15.4%) reported adverse drug reactions [95, 96] and one study found 55.1% experienced a suspected adverse drug reaction [95]. Problems with antidepressants involved dosage errors, drug-drug interactions, drug-disease interactions, and therapeutic duplication [54]. For patients with SMI in a UK study, monitoring of neuroleptics was overdue in 73.0% (16/22) of cases and blood parameters were out of range in 27.0% (6/22) of cases [88].

### Aetiology of drug-related problems

Aetiological data was collected most commonly in studies from the USA (12/25, 48.0%), followed by the UK (5/25, 20.0%), Germany (2/25, 8.0%), Australia (1/25, 4.0%), Belgium (1/25, 4.0%), Canada (1/25, 4.0%), France (1/25, 4.0%), South Africa (1/25, 4.0%), and Scotland (1/25, 4.0%). Out of the 25/79 (31.6%) studies which reported aetiological data, 6/25 (24.0%) were qualitatively derived, 16/25 (64.0%) quantitative, and 3/25 (12.0%) used mixed methods. Data collection methods included interviews/focus groups (12/25, 48.0%), surveys/questionnaires (9/25, 36.0%), medical/pharmacy/medication records (7/25, 28.0%), medication reviews (2/25, 8.0%), and a clinical database (1/25, 4.0%) with seven studies using multiple collection methods. A characteristics summary of the aetiology studies can be seen in Table 3 with a data summary of each study available in Supplementary file 5.

The study populations for the 25 aetiological studies were as follows: patients with mental illness (20/25), general population with mental health subpopulation (4/25), and clinician views (mental health diagnoses unspecified). The full aetiology dataset can be seen in Supplementary file 6. The aetiology of non-adherence was most commonly investigated with 19/25 (76.0%) studies reporting on this DRP. Nineteen studies (19/25, 76.0%) included an objective to research DRP causes and/or risk factors/predictors with fifteen of these studies (15/19, 78.9%) focusing solely on non-adherence and the remaining four studies presenting data for non-adherence and monitoring [113], PIM [107], PIP [103] and PHP/Monitoring [26]. Only two studies [93, 113] provided solely aetiological data, the remaining 23 studies all presented epidemiological data and/or evaluated an intervention as it was their main objective. In total, prescriber-related factors (e.g. time, communication, location) were reported in 8/25 (32.0%) studies and patient-related factors were reported in 23/25 (92.0%) studies. Six aetiological studies 6/25 (24.0%) [54, 55, 88, 93, 106, 113] identified multiple DRPs but only presented aetiological data for a selection of the identified safety challenges.

**Table 3:** Characteristics summary of 25 aetiology studies

Study Characteristic	Characteristic subcategory	No. of studies (%)
Year of Publication	2000–2010	7 (28.0)
	2011 onwards	18 (72.0)
Country of origin	USA	12 (48.0)
	UK*	6 (24.0)
	Europe	4 (16.0)
	Other	3 (12.0)
Primary care setting	General Practice	13 (52.0)
	Community-dwelling	7 (28.0)
	Nursing Homes	3 (12.0)
	Community Pharmacy	2 (8.0)
Data collection method^#^	Interviews/Focus groups	12 (48.0)
	Questionnaire/survey	9 (36.0)
	Medical/Pharmacy/Prescription records	7 (28.0)
	Medication reviews	2 (8.0)
	Clinical database	1 (4.0)
DRP investigated	Non-adherence	19 (76.0)
	PIP/PIM/PHP	4 (16.0)
	ME	4 (16.0)
	Preventable ADR/ADE	1 (4.0)
Aetiology data type	Causal^1^	15 (60.0)
	Risk factor^2^	10 (40.0)
Aetiological factors	Patient-related	23 (92.0)
	Prescriber-related	8 (32.0)

# some studies used multiple methods; * includes Scotland specific study; 1 = Underlying reasons; 2 = Correlations; ADE = Adverse drug event; ADR = Adverse drug reaction; ME = Medication error; PHP = Potentially hazardous prescribing; PIM = Potentially inappropriate medication; PIP = Potentially inappropriate prescribing; UK = United Kingdom; USA = United States of America

#### Risk factor data

The two most common data collection methods were questionnaires (4/10, 40.0%) followed by medical records (2/10, 20.0%). Out of the ten risk factor studies, six reported these for non-adherence [54, 55, 58, 59, 68, 79], one for PIM [86], one for PIP [103], one for PHP and a medication error (monitoring) [26], and one for preventable adverse drug events and a medication error which was drug-drug interactions [106]. Risk factors for non-adherence included patient scepticism about medication (p < 0.05) [58], increased travel time to pharmacies (p = 0.04) [59], and polypharmacy (adjusted relative rate ratio 2.72 (95% CI 1.76–4.21)) [55]. Parsons et al. found a correlation between the number of medications prescribed and the occurrence of PIM (r = 0.335, p < 0.001) [86]. Hiance-Delahaye et al. noted polypharmacy (adjusted OR 5–9 drugs 2.61 (95% CI 1.11– 6.16) and OR ≥ 10 drugs 2.69 (95% CI 1.06–6.87)) and longer symptom duration (adjusted OR 2.82 (95% CI 1.42–6.99)) was correlated with PIP of antidepressants in older patients [103]. A UK study found > 10 repeat prescriptions had a higher risk of PHP (adjusted OR 30.22) but a lower risk of inadequate monitoring (adjusted OR 0.35 (95% CI 0.29–0.41)), and female patients were more at risk of PHP (adjusted OR 1.43 (95% CI 1.41–1.45)) and inadequate monitoring (adjusted OR 1.12 (95% CI 1.05–1.20)) [26]. A study in patients with dementia in Germany found cognitive impairment was associated with preventable adverse drug events (p = 0.004) and a psychiatric diagnosis was associated with inappropriate drug choice (OR 1.66 (95% CI 1.24–2.21) p = 0.001) and therefore adverse drug events (*χ*^2^(10) = 19.38, p = 0.036) and drug-drug interactions (*χ*^2^(10) = 56.15, p < 0.001) [106].

#### Causal data

The most common data collection method used was interviews (9/15, 60.0%) followed by questionnaires (4/15, 26.7%), with the majority of causal data relating to non-adherence (13/15 studies) and the reasons for non-adherence can be viewed in Supplementary file 6. Breakdown of communication was a common theme in two studies [88, 107] with Voigt et al. (mixed methods analysis in Germany) noting that there was poor communication from psychiatrists to GPs regarding prescribed medication and lack of clinical information [107]. However Voigt et al. provided little causal data in the context of mental illness as the main focus of the paper was PIM prescribing in the elderly. Raynsford et al. noted in the 10 cases of drug error discovered by a specialist pharmacist, that 50% of errors were due to poor communication from secondary care and the other 50% were due to GPs not paying attention to instructions from secondary care [88]. Overall however, Raynsford et al. provided little causal data as the main focus was investigating the impact of a specialist mental health pharmacy team within English general practices. All 15 studies presented brief causal data and did not report any incidents arising from multiple contributory factors or wider system involvement.

### Remedial interventions for drug-related problems

Most studies (10/18, 55.6%) adopted the randomised controlled trial study design to evaluate an intervention. The top three countries where interventions were commonly evaluated was the USA (6/18, 33.3%), followed by the UK (4/18), then Slovenia and Australia (2/18 each). The most common setting for intervention studies was general practice (7/18, 38.9%), with 4/18 (22.2%) taking place in community pharmacy, 4/18 (22.2%) in CMHTs, 1/18 (5.6%) in nursing homes, 1/18 (5.6%) in a community mental health clinic, and 1/18 (5.6%) in a non-specific community setting. All of the intervention studies targeted patients with mental illness as the main study population, with one of those studies targeting patients with dementia in general practice specifically [106]. A characteristics summary of the intervention studies can be seen in Table 4. A data summary of each intervention study can be viewed in Supplementary file 7 with the full dataset of interventions and reported outcomes available in Supplementary file 8.

The three most common interventions aimed to improve adherence (11/18, 61.1%) [53, 57, 71, 73, 76, 80, 89, 90, 99, 111, 123], optimise psychotropic drugs (2/18, 11.1%) [88, 119], and evaluate medicine reviews (2/18, 11.1%) [96, 118] with the remainder each evaluating a different intervention.

**Table 4:** Characteristics summary of 18 intervention studies

Study Characteristic	Characteristic subcategory	No. of studies (%)
Year of Publication	2000–2010	4 (22.2)
	2011 onwards	14 (77.8)
Country of origin	USA	6 (33.3)
	UK	4 (22.2)
	Europe	5 (27.8)
	Other	3 (16.7)
Primary care setting	General Practice	7 (38.9)
	Community Pharmacy	4 (22.2)
	CMHT	4 (22.2)
	Nursing Homes	1 (5.6)
	CMHC	1 (5.6)
	Community-dwelling	1 (5.6)
Interventions	Medication review	6 (33.3)
	Education/coaching	3 (16.7)
	Multimodal program	3 (16.7)
	Multidisciplinary teleservice	2 (11.1)
	Financial incentives	2 (11.1)
	Technology	2 (11.1)
DRP targets*	Non-adherence	11 (61.1)
	Nonspecified DRPs	3 (16.7)
	PIM/DDI	2 (11.1)
	Prescribing discrepancies	1 (5.6)
	High dose/multiple antipsychotics	1 (5.6)
	Monitoring	1 (5.6)

* some studies had multiple targets; CMHC = Community mental health clinic; CMHT = Community mental health team; DDI = Drug-drug interaction; DRP = Drug-related problem; PIM = Potentially inappropriate medication; UK = United Kingdom; USA = United States of America

More than half the studies (10/18, 55.6%) utilised a pharmacist to provide an intervention. Eight of the ten pharmacist interventions involved a form of medication review or monitoring service for patients. The remaining two pharmacist interventions were to provide education/coaching to patients to help improve adherence [99, 111]. The DRP targets were non-adherence (11/18), high dose/multiple antipsychotics (1/18), monitoring (1/18), prescribing discrepancies (1/18), PIMs/drug-drug interactions (2/18), and three had non-specified DRP targets with some studies having multiple targets.

Whilst all 18 studies reported positive improvements in some of their measured outcomes (results in Supplementary file 8), 6/18 (33.3%) interventions reported little difference between a few medication safety measures in the intervention and control groups. Outcome measures used in the studies included adherence rates, recommendation approvals, DRP rate reductions, and prescribing discrepancy rates. Rubio-Valera et al. found the intervention group were more likely to remain adherent at 3 and 6 months, however, this did not reach statistical significance [99]. Johnson et al. trialled a three part quality improvement intervention in three CMHTs, and reported that one of the teams achieved a reduced medication discrepancy rate and a non-statistically significant improvement but the other two did not [85]. Raynsford et al. reported a total of 104 interventions for different DRPs such as adherence issues, high dose/multiple antipsychotics, physical health monitoring issues and drug errors. Out of all the interventions made by a specialist medicines optimisation team, 5.8% were graded as hospital admission prevented and 23.1% were graded as being of no clinical significance to the patient [88]. Priebe et al. (2016) trialled stopping financial incentives in UK antipsychotic users and found no statistically significant difference in mean adherence between the control and intervention group [89]. Corden et al. trialled a mobile app in the USA to improve adherence, adherence was 88.5% during the first four weeks, and in the final four weeks of the eight week trial there was a non-statistical decrease in adherence to 73.0% ^(76)^. Finally, Brook et al. evaluated a pharmacy coaching program for patients in the Netherlands and the intention-to-treat analyses showed no effect on adherence. Only the analysis of patients who received per-protocol interventions had statistically significant better adherence in the intervention group compared to controls (95% CI 5.1–28.9, p < 0.05) [111].

## Discussion

To our knowledge this is the first study to identify, gather and describe the global published literature concerning the epidemiology, aetiology, and impact of remedial interventions designed to improve medication safety in populations with mental illness within a primary care setting. A total of 79 studies were identified, with 77 reporting on epidemiology, 25 on aetiology, and 18 evaluated interventions. This review has highlighted important medication safety challenges that patients with mental illness may face in primary care, and supports the WHO and NHS in having medicines safety [1, 2] and mental health patient safety [2] as key areas for improvement. The DRPs investigated most commonly were non-adherence followed by PIP/PIM/PHP with very little aetiological and intervention data reported for other preventable medication safety challenges. Depression was the most commonly cited diagnosis overall and a common focus in medication error/adverse drug event studies. Patients with dementia or elderly with mental illness comorbidities made up the majority of PIP/PIM studies. Schizophrenia and bipolar disorder were mainly represented in non-adherence studies and patients with other diagnoses such as personality disorders and attention deficit hyperactivity disorder were underrepresented as a whole. The majority of interventions involved a specialist pharmacist and most examined interventions designed to improve adherence rates to psychotropics; whilst many reported positive results on some medication safety measures, some measures reported little difference between groups.

This review highlighted that the epidemiology, aetiology, and trialled interventions of non-adherence have been extensively explored within primary care. Some non-adherence rates reported were considerably higher than other patient groups with chronic conditions in primary care [130]. Patients commonly reported side effects as a major contributor to their non-adherence, a key influencing factor in another systematic review [131]. Non-adherence is a critical issue in mental health care as it can lead to illness exacerbation and reduced efficacy of treatments [131]. The methodologies used mostly sought information from one source which may have limited the exploration of other contributory factors and wider system involvement, and might explain why patient-related factors were the most cited cause. Depression was the most common diagnosis within the non-adherence studies and further insights are required for other mental health conditions and associated medications. Commonly intervention studies evaluated a single novel intervention such as using technology or educational programs which demonstrated some success, however, as this review as well as another [132] found, the causes of non-adherence are multifactorial, and therefore likely require multifaceted intervention. Recommendations for future policy development regarding non-adherence are needed as it has been clearly evidenced as a prevalent and well-understood issue.

The epidemiology and risk factors for PIP/PIM was the second most commonly researched safety challenge but not a common target for remedial intervention studies. The majority of focus for PIP/PIM studies was older patients and those with dementia based in nursing homes. Older patients are at risk of medication safety events as this population are likely to have comorbidities, polypharmacy [133] and an increased risk of using inappropriate medication [134]. It is known that older patients are subject to metabolic changes, reduced clearance, and polypharmacy which can lead to drug-drug interactions [134]. Whilst it is therefore understandable that PIP/PIM studies have focused on older patients, a focus on those with specific diagnoses or in particular settings may not provide a generalisable view across all patients with mental illness. One recent study by Khawagi et al. highlighted the prevalence and risk factors for potentially hazardous prescribing in broader populations with mental illness in UK primary care, and may be used to guide further international work [26]. Aetiological data from three studies cited communication and polypharmacy as key issues but overall was limited to specific patient groups and contexts which may adversely affect the development of remedial interventions. These findings could be useful for future intervention research as they identify some key mental health patient groups where PIP/PIM is prevalent for development of targeted interventions. Electronic health record searches (such as a pharmacist-led information technology intervention for medication errors (PINCER) and the Salford medication safety dashboard (SMASH)) have been trialled as potential interventions for PIP at scale in primary care [135, 136] and demonstrated success as they can be applied on a large scale, promote multidisciplinary working, and involve medicines experts such as pharmacists. There may therefore be scope for a similar PIP/PIM intervention for patients with mental illness [26], with limited evidence from PIP/PIM intervention studies in this review also suggesting benefits of multidisciplinary working and specialist mental health pharmacist reviews. The findings that PIP/PIM are prevalent issues supports the delivery of training and implementation of electronic prescribing systems to reduce prescribing error rates and improve safety of prescriptions [2] which have demonstrated reductions in prescribing errors in other settings such as hospitals [137].

This review has emphasised the positive impact specialist mental health pharmacists can have on the care of patients with mental illness in primary care, including medication reviews, timely follow-ups, and appropriate drug choice/prescribing. This finding is consistent with other research which suggests pharmacists could provide integrated care, follow-ups, and evidence-based pharmacotherapy [138, 139]. This review also supports NHS plans to expand the number of clinical pharmacists in primary care to provide medicine reviews [140] and to train more specialist mental health pharmacists for roles in primary care such as working in CMHTs [141], ultimately improving efficiency and patient care. Specialist pharmacist medication reviews are supported by this review and are involved in the NHS Long Term Plan [140]. Pharmacists were also integral in the data collection of included studies which highlights the importance of their role in identifying and measuring the quality and safety of medication use.

The majority of the causal and risk factor data was for non-adherence and other DRPs were poorly represented. Risk factor data identified correlations but not causation so our understanding of the aetiology for other DRPs such as PIP/PIM, medication errors and preventable adverse drug events remains limited. The main aetiological findings for non-adherence were mostly related to patient factors such as side effects. Prescriber-related factors are less commonly reported despite evidence in this review of safety challenges originating from prescriber such as potentially inappropriate prescribing. Limited risk factor and causal data for DRPs such as PIP/PIM, medication errors, and preventable adverse drug events means there is less knowledge to guide the development of theory-driven and targeted interventions that have the best chance of success. Using theory-driven approaches when designing patient safety interventions helps to demonstrate feasibility and acceptability [142]. Communication difficulties between services were highlighted in this review as a contributor to preventable medication safety incidents. This supports the need for future research regarding care transitions, which the WHO have also identified as a key challenge - *medication safety in transitions of* care [143].

Our findings reveal that other DRP outcomes such as medication errors and preventable adverse drug events have received little attention in the literature and there was a common focus on patients with depression and elderly patients in medication error and adverse drug event studies. The existing data does not provide a clear epidemiological and aetiological picture as the relevant studies did not focus on the aetiology of these DRPs as their primary objective, and/or included a small variety of medication errors and adverse drug events; thus the prevalence and causes of a wider range of error sub-types such as prescribing, monitoring, dispensing and administration have yet to be established. It is important to target medication errors and related adverse drug events as these events are inherently preventable and cost the NHS just over £98 million per year [29], hence why they are targets for national and international policies [1, 2]. There is evidence from studies in other disease states such as diabetes and congestive heart failure, that pharmacist-led medication reviews could identify and prevent adverse drug events bringing considerable cost savings [144]. Recent evidence has highlighted that medication errors and adverse drug events are common in secondary care mental health settings and have unique factors which underpin their nature and aetiology [18, 19]; the available evidence from primary care in this review does not confirm whether these same factors apply or not and so this question must be explored in-depth as seen elsewhere in this setting [145] to guide the development of targeted interventions with the best chance of success.

### Strengths and limitations

This is the first study to compile available evidence concerning the epidemiology, aetiology, and interventions for DRPs in patients with mental illness in primary care. A systematic approach was followed to develop and complete the search which allowed transparency and rigour. Independent extraction of study characteristics and data was carried out by two additional reviewers. However, there were several limitations to the review. The screening was carried out by one reviewer (MJA) which may have resulted in studies being inappropriately excluded however, any cases of uncertainty were discussed amongst the research team. Grey literature was excluded so some additional insights may be missing. Quality assessment of the studies was not carried out as it can lead to a form of selection bias [146] and also the aim of the review was to primarily explore all of the available peer-reviewed literature and identify future research targets. Non-english language studies were excluded which may have led to exclusion of relevant non-English studies. If a study reported on one specific drug or error subtype then it was excluded, and whilst including this data may lead to additional insights it would not support generalisability across populations with mental illness which was the purpose of this review. The term “mental illness” is relatively broad with no unified accurate definition [147, 148] which can lead to significant heterogeneity between how each study defines the population. This difficulty was minimised by including studies which confirmed their study population by diagnosis or formal screening and search terms within the strategy encompassed multiple variations of the term.

## Conclusion

This is the first scoping review to identify and describe published literature concerning medication safety challenges for patients with mental illness in primary care. It revealed that medication safety challenges are common and that non-adherence and PIP/PIM are the most commonly investigated types of drug-related problem. The available data identified important targets that may form the focus of future interventions. Compared to these outcomes, there is currently limited epidemiological and aetiological data regarding medication errors and preventable adverse drug events for those with mental illness in primary care settings, with gaps across outcomes for specific mental health diagnoses and wider patient age groups. Future work should further explore the epidemiology and aetiology of medication errors and preventable adverse drug events across wider groups of patients with mental illness. This scoping review can be used to inform future work on the pathway to developing remedial interventions to improve the outcomes for patients with mental illness.

### Electronic supplementary material

Below is the link to the electronic supplementary material.

Supplementary Material 1 - Embase search strategy

Supplementary Material 2 - Data extraction form

Supplementary Material 3 - Dataset summary of 79 included studies

Supplementary Material 4 - Data summary of 77 epidemiology studies

Supplementary Material 5 - Data summary of 25 aetiology studies

Supplementary Material 6 - Aetiology of medication safety challenges full dataset

Supplementary Material 7 - Data summary of 18 intervention studies

Supplementary Material 8 - Intervention studies and reported outcomes full dataset

Supplementary Material 9 - PRISMA-ScR Checklist

## Data Availability

The data generated and analysed during this study to support the findings are included in this published article and its supplementary files. Any additional datasets used and/or analysed during the current study are available from the corresponding author on reasonable request.

## Abbreviations

CMHT: Community Mental Health Team
CVD: Cardiovascular Disease
DRP: Drug-related problem
GP: General Practitioner
NHS: National Health Service
PHP: Potentially Hazardous Prescribing
PIM: Potentially Inappropriate Medication
PINCER: Pharmacist-led information technology intervention
PIP: Potentially Inappropriate Prescribing
PRISMA-ScR: Preferred Reporting Items for Systematic Reviews and Meta-Analyses Scoping Review
SMASH: Salford medication safety dashboard
SMI: Serious Mental Illness
UK: United Kingdom
USA: United States of America
WHO: World Health Organization
