# Supplementary material for: Understanding the medication safety challenges for patients with mental illness in primary care: a scoping review
Source: BMC Psychiatry. 2023 Jun 12;23:417. doi: 10.1186/s12888-023-04850-5 (PMC10258931; doi:10.1186/s12888-023-04850-5)
Supplement: Supplementary file 1 — Supplementary Material 1 - Embase search strategy [file 12888_2023_4850_MOESM1_ESM.docx]

**Embase search strategy**

1 mental health/

2 mental disorder.mp. or mental disease/

3 mental illness.mp.

4 "mixed anxiety and depression"/ or depression/

5 anxiety/ or anxiety disorder/

6 psychosis/ or schizo*.mp.

7 bipolar disorder/ or bipolar depression/ or bipolar.mp.

8 dementia/

9 1 or 2 or 3 or 4 or 5 or 6 or 7 or 8

10 general practice/

11 primary care.mp.

12 nursing home/ or care home.mp. or elderly care/

13 primary medical care/ or general practitioner/ or GP practice.mp.

14 community pharmacy.mp.

15 ambulatory care/

16 10 or 11 or 12 or 13 or 14 or 15

17 drug safety/ or medication safety.mp. or medication error/ or patient safety/

18 adverse drug event.mp. or adverse drug reaction/

19 prescribing error.mp.

20 medication adherence.mp. or medication compliance/

21 non-adherence.mp.

22 administration error.mp.

23 dispensing error.mp.

24 17 or 18 or 19 or 20 or 21 or 22 or 23

25 9 and 16 and 24

26 limit 25 to (english and yr="2000 - Current")
