# Supplementary material for: Understanding the medication safety challenges for patients with mental illness in primary care: a scoping review
Source: BMC Psychiatry. 2023 Jun 12;23:417. doi: 10.1186/s12888-023-04850-5 (PMC10258931; doi:10.1186/s12888-023-04850-5)
Supplement: Supplementary file 2 — Supplementary Material 2 - Data extraction form [file 12888_2023_4850_MOESM2_ESM.docx]

**Standardised data extraction form**

| Title, Authors, Year | Country | Setting | Study period | Study design | Study population | Aim of study | Outcomes measured | Definitions of terms used e.g. adherence | Data validation methods |
| --- | --- | --- | --- | --- | --- | --- | --- | --- | --- |
|  |  |  |  |  |  |  |  |  |  |

| Data collection methods | Personnel collecting data | Epidemiology of errors | Aetiology of errors | Preventable harm caused | Interventions | Limitations |
| --- | --- | --- | --- | --- | --- | --- |
|  |  |  |  |  |  |  |
