# Supplementary material for: Understanding the medication safety challenges for patients with mental illness in primary care: a scoping review
Source: BMC Psychiatry. 2023 Jun 12;23:417. doi: 10.1186/s12888-023-04850-5 (PMC10258931; doi:10.1186/s12888-023-04850-5)
Supplement: Supplementary file 4 — Supplementary Material 4 - Data summary of 77 epidemiology studies [file 12888_2023_4850_MOESM4_ESM.docx]

| **Author & Year** | **Country** | **Primary care setting** | **Relevant psychiatric diagnoses within study population** | **Data collection method** | **Drug-related problem** |
| --- | --- | --- | --- | --- | --- |
| J. S. Bell et al. (2006) | Australia | Pharmacy | ●Psychosis  ●Depression  ●Anxiety disorder | Medication review | ●Non-adherence  ●PIM  ●ME  ●ADR |
| N. Gisev et al. (2010) | Australia | CMHT | ●Bipolar disorder  ●Psychosis  ●Depression  ●Anxiety disorder | Medication review | ●Non-adherence  ●PIM  ●ME  ●ADR |
| S. L. Harrison et al. (2018) | Australia | Nursing home | Dementia | ●Pharmacy records  ●Medication charts  ●Questionnaire | PIM |
| S. C. Woodward et al. (2016) | Australia | Community-dwelling | Depression | Online survey | Non-adherence |
| K. Demyttenaere et al. (2001) | Belgium | Community-dwelling | Depression | ●Questionnaire  ●Interview | Non-adherence |
| A. I. Miasso et al. (2016) | Brazil | GP practice* | NS | ●Questionnaire  ●Interview | Non-adherence |
| S. N. Rezansoff et al. (2016) | Canada | Community-dwelling | ●Bipolar disorder  ●Psychosis  ●NS | Prescription records | Non-adherence |
| L. Dou et al. (2020) | China | Community-dwelling | ●Bipolar disorder  ●Psychosis | Questionnaire | Non-adherence |
| D. Fialová et al. (2005) | Europe – 8 countries | Community-dwelling | Depression | ●Interview  ●Medical records | ●Non-adherence  ●PIM |
| H. M. Hosia-Randell et al. (2008) | Finland | Nursing home | ●Dementia  ●Depression | Medical charts | ●PIM  ●ME |
| A. Hiance-Delahaye et al. (2018) | France | Community-dwelling | Depression | Interview | PIP |
| C. Cool et al. (2014) | France | Nursing home | ●Dementia  ●Depression  ●Other^#^ | Prescription records | PIP |
| C. Laffon de Mazières et al. (2015) | France | Nursing home | ●Dementia  ●Bipolar disorder  ●Depression | Prescription records | Inappropriate neuroleptics (PIP) |
| D. Wucherer et al. (2017) | Germany | GP practice | ●Dementia  ●Depression | Medication review | ●Non-adherence  ●PIM  ●ME  ●ADE |

**Data summary of 77 epidemiology studies**

| **Author & Year** | **Country** | **Primary care setting** | **Relevant psychiatric diagnoses within study population** | | **Data collection method** | | **Drug-related problem** |  |
| --- | --- | --- | --- | --- | --- | --- | --- | --- |
| K. Voigt et al. (2016) | Germany | GP practice | NS | ●Medical records  ●Interview | | PIM | |  |
| A. Booker et al. (2016) | Germany | GP practice | Dementia | Clinical database | | Non-adherence | |  |
| A. Pillai et al. (2021) | India | GP practice* | Depression | Interview | | Non-adherence | |  |
| S. B. Sriramulu et al. (2021) | India | CMHC | ●Bipolar disorder  ●Psychosis  ●Depression  ●Anxiety disorder  ●Other^#^ | Medical records | | Non-adherence | |  |
| L. Ayalon et al. (2011) | Israel | GP practice | ●Depression  ●Anxiety disorder | ●Clinical database  ●Pharmacy records | | Non-adherence | |  |
| E. C. van Geffen et al. (2009) | | Netherlands | GP practice | ●Depression  ●Anxiety disorder | ●Clinical database  ●Pharmacy records | | Non-adherence | |
| F. Holvast et al. (2018) | | Netherlands | GP practice | Depression | ●Clinical database  ●Pharmacy records | | Non-adherence | |
| O. H. Brook et al. (2005) | | Netherlands | Pharmacy | Depression | ●Pharmacy records  ●Electronic pill counter | | Non-adherence | |
| D. Lee et al. (2013) | | New Zealand | Community-dwelling | Depression | ●Interview  ●Home medication recorded | | PIM | |
| C. Burton et al. (2012) | | Scotland (UK) | GP practice | Depression | Clinical database | | Non-adherence | |
| M. Jaffray et al. (2014) | | Scotland (UK) | GP practice | Depression | Interview | | Non-adherence | |
| M. Stuhec et al. (2019) | | Slovenia | Nursing home | NS | ●Medical charts  ●Medication review | | ●PIM  ●ME | |
| M. Stuhec et al. (2021) | | Slovenia | GP practice | ●Dementia  ●Psychosis  ●Depression  ●Anxiety disorder | ●Medical charts  ●Medication review | | PIM | |
| L. Kalimashe et al. (2021) | | South Africa | GP practice* | NS | Questionnaire | | Non-adherence | |
| M. Rubio-Valera et al. (2013) | | Spain | Pharmacy | Depression | Pharmacy records | | Non-adherence | |
| I. Aznar-Lou et al. (2018) | | Spain | GP practice | Depression | Clinical database | | Non-adherence | |
| M. J. Serrano et al. (2014) | | Spain | GP practice* | Depression | Questionnaire | | Non-adherence | |
| S. Oller-Canet et al. (2011) | | Spain | GP practice* | ●Depression  ●Anxiety disorder | Medical records | | Non-adherence | |
| C. Freccero et al. (2016) | | Sweden | Pharmacy | ●Depression  ●Anxiety disorder | Clinical database | | Non-adherence | |
| T. C. Wang et al. (2019) | | Taiwan (ROC) | Pharmacy | NS | Pharmacy records | | PIM | |

| **Author & Year** | **Country** | **Primary care setting** | **Relevant psychiatric diagnoses within study population** | **Data collection method** | **Drug-related problem** |  |
| --- | --- | --- | --- | --- | --- | --- |
| C. F. Johnson et al. (2020) | UK | CMHT | ●Bipolar disorder  ●Psychosis  ●Depression  ●Anxiety disorder  ●Other^#^ | Medical records | ME |  |
| C. Parsons et al. (2012) | UK | Nursing home | Dementia | Medication administration records | PIM |  |
| J. Delgado et al. (2021) | UK | GP practice | ●Dementia  ●Depression | Clinical database | PIP |  |
| J. Raynsford et al. (2020) | | UK | GP practice | NS | Medical records | ●Non-adherence  ●ME |
| W. Y. Khawagi et al. (2021) | | UK | GP practice | NS | Clinical database | ●PHP  ●ME |
| S. Priebe et al. (2016) | | UK | CMHT | ●Bipolar disorder  ●Psychosis | Medical records | Non-adherence |
| S. Priebe et al. (2013) | | UK | CMHT | ●Bipolar disorder  ●Psychosis | Medical records | Non-adherence |
| M. Falcaro et al. (2019) | | UK | GP practice | ●Dementia  ●Depression | Clinical database | Non-adherence |
| P. Saini et al. (2018) | | UK | GP practice | ●Psychosis  ●Depression  ●Anxiety disorder  ●Other^#^ | ●Medical records  ●Interview | Non-adherence |
| S. Davé et al. (2012) | | UK | GP practice | ●Depression  ●Anxiety disorder | Clinical database | Non-adherence |
| D. G. Stevenson et al. (2010) | | USA | Nursing home | ●Dementia  ●Depression | ●Medical records  ●Interview | PIM |
| J. C. Fortney et al. (2011) | | USA | GP practice | Depression | ●Pharmacy records  ●Interview | Non-adherence |
| J. T. Hanlon et al. (2011) | | USA | Nursing home | ●Dementia  ●Bipolar disorder  ●Psychosis  ●Depression  ●Anxiety disorder | ●Medical records  ●Interview | ●Non-adherence  ●ME |
| J. T. Hanlon et al. (2015) | | USA | Nursing home | ●Dementia  ●Bipolar disorder  ●Psychosis  ●Depression  ●Anxiety disorder | ●Medical records  ●Pharmacy records | ●Non-adherence  ●PIP  ●ME |
| T. G. Rhee et al. (2018) | | USA | Ambulatory care | ●Dementia  ●Bipolar disorder  ●Psychosis  ●Depression  ●Anxiety disorder  ●Other^#^ | Questionnaire | PIM |

| **Author & Year** | **Country** | **Primary care setting** | **Relevant psychiatric diagnoses within study population** | **Data collection method** | **Drug-related problem** |  |
| --- | --- | --- | --- | --- | --- | --- |
| J. A. Sirey et al. (2017) | USA | GP practice* | Depression | Questionnaire | Non-adherence |  |
| J. E. Aikens et al. (2005) | USA | GP practice* | Depression | Questionnaire | Non-adherence |  |
| P. H. Noel et al. (2005) | USA | GP practice* | ●Depression  ●Anxiety disorder | Questionnaire | Non-adherence |  |
| J. J. Stephenson et al. (2012) | | USA | Community-dwelling | ●Bipolar disorder  ●Psychosis | ●Clinical database  ●Survey | Non-adherence |
| T. P. Gilmer et al. (2004) | | USA | Pharmacy | Psychosis | Pharmacy records | Non-adherence |
| D. Ganoczy et al. (2007) | | USA | Community-dwelling | Bipolar disorder | Clinical database | Non-adherence |
| M. B. Tamburrino et al. (2009) | | USA | GP practice* | Depression | Questionnaire | Non-adherence |
| S. C. Marcus et al. (2015) | | USA | Community-dwelling | ●Bipolar disorder  ●Psychosis  ●Depression  ●Anxiety disorder | Clinical database | Non-adherence |
| F. Forma et al. (2020) | | USA | Community-dwelling | ●Bipolar disorder  ●Psychosis  ●Depression | Clinical database | Non-adherence |
| M. Olfson et al. (2006) | | USA | Community-dwelling | Depression | Interview | Non-adherence |
| J. F. Farley et al. (2011) | | USA | Community-dwelling | Psychosis | Clinical database | Non-adherence |
| J. A. Bates et al. (2010) | | USA | Community-dwelling | Bipolar disorder | Questionnaire | Non-adherence |
| R. Nayak et al. (2019) | | USA | Community-dwelling | Psychosis | Clinical database | Non-adherence |
| D. C. Bultman et al. (2000) | | USA | Pharmacy | Depression | Interview | Non-adherence |
| S. Bhat et al. (2018) | | USA | GP practice* | Depression | Medication review | Non-adherence |
| C. A. Fontanella et al. (2011) | | USA | Community-dwelling | ●Depression  ●Anxiety disorder  ●Other^#^ | Clinical database | Non-adherence |
| L. Hoffman et al. (2003) | | USA | Pharmacy | Depression | Pharmacy records | Non-adherence |
| S. Singhal et al. (2021) | | USA | GP practice* | NS | Medical records | ME |

| **Author & Year** | **Country** | **Primary care setting** | **Relevant psychiatric diagnoses within study population** | **Data collection method** | **Drug-related problem** |
| --- | --- | --- | --- | --- | --- |
| B. Pyenson et al. (2013) | USA | Community-dwelling | Psychosis | Clinical/insurance database | Non-adherence |
| M. E. Corden et al. (2016) | USA | GP practice* | Depression | ●Questionnaire  ●Electronic pill counter | Non-adherence |
| H. C. Kales et al. (2016) | USA | GP practice* | ●Bipolar disorder  ●Psychosis  ●Depression  ●Anxiety disorder  ●Other^#^ | ●Questionnaire  ●Interview  ●Pharmacy records | Non-adherence |
| M. Sajatovic et al. (2009) | USA | CMHC | Bipolar disorder | Questionnaire | Non-adherence |
| M. Spoont et al. (2005) | USA | GP practice* | Anxiety disorder | Questionnaire | Non-adherence |
| D. Velligan et al. (2013) | USA | CMHC | Psychosis | Electronic pill counter | Non-adherence |
| S. L. Toomey et al. (2012) | USA | Community-dwelling | Other^#^ | Interview | Non-adherence |
| M. Dibonaventura et al. (2012) | USA | Community-dwelling | Psychosis | ●Questionnaire  ●Interview | Non-adherence |
| M. Sajatovic et al. (2006) | USA | Community-dwelling | ●Bipolar disorder  ●Anxiety disorder | Clinical database | Non-adherence |
| M. Sajatovic et al. (2007) | USA | Community-dwelling | ●Bipolar disorder  ●Anxiety disorder | Clinical database | Non-adherence |

# Such as personality disorders, attention deficit hyperactivity disorder; *Setting interpreted as general practice; ADE = Adverse drug event; ADR = Adverse drug reaction; CMHC = Community Mental health Clinic; CMHT = Community Mental Health Team; ME = Medication error; NS = Nonspecified; PIM = Potentially Inappropriate Medication; PIP = Potentially Inappropriate Prescribing; ROC = Republic of China; UK = United Kingdom; USA = United States of America
