# Supplementary material for: Understanding the medication safety challenges for patients with mental illness in primary care: a scoping review
Source: BMC Psychiatry. 2023 Jun 12;23:417. doi: 10.1186/s12888-023-04850-5 (PMC10258931; doi:10.1186/s12888-023-04850-5)
Supplement: Supplementary file 5 — Supplementary Material 5 - Data summary of 25 aetiology studies [file 12888_2023_4850_MOESM5_ESM.docx]

**Data summary of 25 aetiology studies**

| **Author & Year** | **Country** | **Data type presented & collection method** | **Drug-related problem** | **Aetiology data type** |
| --- | --- | --- | --- | --- |
| D. Wucherer et al. (2017) | Germany | Quantitative – medication review | ●ADE  ●ME (DDI) | Risk factor |
| S. C. Woodward et al. (2016) | Australia | Quantitative – online survey | Non-adherence | Causal |
| M. Jaffray et al. (2014) | Scotland (UK) | Qualitative – interview | Non-adherence | Causal |
| K. Demyttenaere et al. (2001) | Belgium | Mixed – questionnaire & telephone interview | Non-adherence | Causal |
| L. Guillaumie et al. (2015) | Canada | Qualitative – focus group | ●Non-adherence  ●ME (Monitoring) | Causal |
| L. Kalimashe et al. (2021) | South Africa | Quantitative – questionnaire | Non-adherence | Causal |
| P. Saini et al. (2018) | UK | Qualitative – medical records & interview | Non-adherence | Causal |
| I. D. Maidment et al. (2017) | UK | Qualitative – interview | Non-adherence | Causal |
| J. C. Fortney et al. (2011) | USA | Mixed – pharmacy records & interview | Non-adherence | Causal |
| J. E. Aikens et al. (2005) | USA | Quantitative - questionnaire | Non-adherence | Risk factor |
| P. H. Noel et al. (2005) | USA | Quantitative - questionnaire | Non-adherence | Risk factor |
| M. B. Tamburrino et al. (2009) | USA | Quantitative - questionnaire | Non-adherence | Causal |
| J. A. Bates et al. (2010) | USA | Quantitative - questionnaire | Non-adherence | Risk factor |
| D. C. Bultman et al. (2000) | USA | Qualitative - interview | Non-adherence | Causal |
| S. Bhat et al. (2018) | USA | Quantitative – medication review | Non-adherence | Causal |
| M. Spoont et al. (2005) | USA | Quantitative - questionnaire | Non-adherence | Risk factor |
| S. L. Toomey et al. (2012) | USA | Qualitative – telephone interview | Non-adherence | Causal |
| M. Dibonaventura et al. (2012) | USA | Quantitative – questionnaire & interview | Non-Adherence | Causal |
| J. T. Hanlon et al. (2011) | USA | Quantitative – medical records & interview | Non-adherence | Risk factor |
| J. T. Hanlon et al. (2015) | USA | Quantitative – medical/pharmacy records | Non-adherence | Risk factor |
| J. Raynsford et al. (2020) | UK | Quantitative – medical records | ME (Monitoring,  Drug errors – wrong dose, unclear directions) | Causal |
| C. Parsons et al. (2012) | UK | Quantitative – medication administration records | PIM | Risk factor |
| W. Y. Khawagi et al. (2021) | UK | Quantitative – Clinical database | PHP ME (Monitoring) | Risk factor |
| K. Voigt et al. (2016) | Germany | Mixed – interview & patient medical records | PIM | Causal |
| A. Hiance-Delahaye et al. (2018) | France | Quantitative - interview | PIP | Risk factor |

AD = Antidepressant; ADE = Adverse drug event; ADHD = Attention deficit hyperactivity disorder; DDI = Drug-Drug interaction; ME = Medication error; PHP = Potentially Hazardous Prescribing; PIM = Potentially inappropriate medication; PIP = Potentially inappropriate prescribing; UK = United Kingdom; USA = United States of America
