# Supplementary material for: Understanding the medication safety challenges for patients with mental illness in primary care: a scoping review
Source: BMC Psychiatry. 2023 Jun 12;23:417. doi: 10.1186/s12888-023-04850-5 (PMC10258931; doi:10.1186/s12888-023-04850-5)
Supplement: Supplementary file 6 — Supplementary Material 6 - Aetiology of medication safety challenges full dataset [file 12888_2023_4850_MOESM6_ESM.docx]

Aetiology of medication safety challenges full dataset

| **Author & Year** | **Data type & Collection method** | **Safety challenge(s) with aetiological data** | **Aetiology of safety challenge(s)** | |
| --- | --- | --- | --- | --- |
|  |  |  | **Prescriber-related** | **Patient-related** |
| D. Wucherer et al. (2017)* | Quantitative – medication reviews | ADE  DDI | **-** | ●Cognitive impairment was associated with ADEs (OR: 1.20; 95% CI: 1.06–1.36; *p* = 0.004).  ●Total number of drugs taken (OR: 1.26; 95% CI: 1.15–1.39; *p* < 0.001) and support with medication (OR: 1.78; 95% CI: 1.05–3.02; *p* = 0.033) were associated with drug interactions.  ●Psychiatric diagnosis associated with problems of inappropriate drug choice (OR: 1.66; 95% CI: 1.24–2.21; *p* = 0.001; significant regression model for problems with inappropriate drug choice: χ2(10) = 33.30, *p*<0.001; problems with ADEs: χ2(10) = 19.38, *p* = 0.036; problems with interactions: χ2(10) = 56.15, *p*<0.001).  ●Total number of drugs taken (b = 0.07; 95% CI: 0.05–0.09; *p*<0.001) and a mental illness (b = 0.09; 95% CI: 0.03–0.15; *p* = 0.003) were associated with total number of DRPs (significant regression model: F (11,89) = 6.18, *p*<0.001) |
| S. C. Woodward et al. (2016) | Quantitative – online survey | Non-adherence | **-** | ●Worried about possible adverse effects  ●Experienced adverse effect(s)  ●Did not think needed to be on an antidepressant  ●Feeling better  ●Lack of efficacy  ●Fear of dependence |
| M. Jaffray et al. (2014) | Qualitative - interviews | Non-adherence | Perceived level of support from GP | ●Knowledge of depression & treatments  ●The level of responsibility patients had for the  management of their depression  ●Perceived level of support from family & friends |

| **Author & Year** | **Data type & Collection method** | **Safety challenge(s) with aetiological data** | **Aetiology of safety challenge(s)** | |
| --- | --- | --- | --- | --- |
|  |  |  | **Prescriber-related** | **Patient-related** |
| K. Demyttenaere et al. (2001) | Mixed – questionnaire & telephone interviews | Non-adherence | **-** | ●Feeling better  ●Adverse events  ●Fear of dependence  ●Feeling uncomfortable taking drugs  ●Lack of efficacy  ●Belief that should solve problems without drugs  ●GP told me to stop |
| L. Guillaumie et al. (2015) | Qualitative – focus groups | Non-adherence | **-** | Side effects |
| L. Kalimashe et al. (2021) | Quantitative - questionnaire | Non-adherence | **-** | ●Forgot to take  ●Careless when taking medication  ●Feeling better  ●Feeling worse  ●Take only when sick  ●Feeling that it is unnatural for mind and body to be controlled by medication  ●Feeling like a ‘zombie’  ●Side effects – tired and sluggish |
| P. Saini et al. (2018) | Qualitative – medical records & interviews | Non-adherence | ●Lack of treatment choices  ●Availability of services for comorbidity and dual diagnosis | Anosognosia |
| I. D. Maidment et al. (2017) | Qualitative - interviews | Non-adherence | ●Patient lack of access to prescribers  ●Lack of time for medication reviews | ●Cognitive impairment  ●Polypharmacy |

| **Author & Year** | **Data type & Collection method** | **Safety challenge(s) with aetiological data** | **Aetiology of safety challenge(s)** | |
| --- | --- | --- | --- | --- |
|  |  |  | **Prescriber-related** | **Patient-related** |
| J. C. Fortney et al. (2011) | Mixed – pharmacy records & interviews | Non-adherence | **-** | ●Never started AD: Concerned about side effects, should solve problems without ADs, afraid would feel different, ADs not safe, ADs won't help  ●Discontinued AD: Not helping, side effects, felt different, should solve problems without ADs, not someone who takes ADs  ●Not taking as prescribed: Forgot to take dose, taking AD on PRN basis, concerned about addiction, side effects, not helping, should solve without ADs, not someone who takes ADs, forgot to refill prescription |
| J. E. Aikens et al. (2005)* | Quantitative - questionnaire | Non-adherence | **-** | ●Patients who were sceptical about medication were significantly less adherent to treatment than both ambivalent and accepting patients (both *p*<0.05)  ●Difference from indifferent patients not statistically significant (*p* = 0.057) |
| P. H. Noel et al. (2005)* | Quantitative - questionnaire | Non-adherence | **-** | ●Travel time to a pharmacy of less than 30 minutes were more likely to be adherent at 1 month (*p =* 0.04)  ●Travel time of less than 30 minutes continued  to predict adherence at 6 months (*p =* 0.01) |
| M. B. Tamburrino et al. (2009) | Quantitative - questionnaire | Non-adherence | **-** | ●Felt better or worse *p*<0.001  ●Careless about taking their medication *p*<0.001  ●Forgotten to take their medication *p*<0.05  ●Believed the medication may harm them *p*<0.001  ●Worried about side effects *p*<0.01  ●Had asked the doctor for an antidepressant medication *p*<0.05  ●Had requested a specific antidepressant *p*<0.01 |

| **Author & Year** | **Data type & Collection method** | **Safety challenge(s) with aetiological data** | **Aetiology of safety challenge(s)** | |
| --- | --- | --- | --- | --- |
|  |  |  | **Prescriber-related** | **Patient-related** |
| J. A. Bates et al. (2010)* | Quantitative - questionnaire | Non-adherence | **-** | ●Significant negative correlations between the symptom severity (OR = 0.812, 95% CI, 0.665–0.992) and the side effect scales (OR = 0.962, 95% CI, 0.937–0.987) on adherence |
| D. C. Bultman et al. (2000) | Qualitative - interview | Non-adherence | ●Physician initial collaborative communication style | ●Initial beliefs/Knowledge  ●Treatment satisfaction |
| S. Bhat et al. (2018) | Quantitative – medication review | Non-adherence | **-** | ●Higher number of prescription medications associated with nonadherence and adverse effects |
| M. Spoont et al. (2005)* | Quantitative - questionnaire | Non-adherence | **-** | ●Biologic conceptualisation associated with nearly a fivefold increased likelihood of underuse of medication  ●Benzodiazepine use |
| S. L. Toomey et al. (2012) | Qualitative – telephone interview | Non-adherence | **-** | ●ADHD is best treated with counselling  ●Preference for counselling over medicine to treat child’s ADHD  ●Belief that medicines to treat ADHD have bad side effects  ●Belief that sometimes do not need to use as much ADHD medicine as the doctor has prescribed  ●Worries about the long-term effects of ADHD medication  ●Perceived lack of medication effectiveness |
| M. Dibonaventura et al. (2012) | Quantitative – questionnaire & interview | Non-Adherence | **-** | Side effects assessed were significantly associated with a decreased likelihood of medication adherence |

| **Author & Year** | **Data type & Collection method** | **Safety challenge(s) with aetiological data** | **Aetiology of safety challenge(s)** | |
| --- | --- | --- | --- | --- |
|  |  |  | **Prescriber-related** | **Patient-related** |
| J. T. Hanlon et al. (2011)* | Quantitative – medical records & interviews | Overuse/underuse (non-adherence) | **-** | ●Factors significantly associated with a reduced risk of potential underuse in patients with depression included polypharmacy (i.e., taking > 5 medications), history of cancer, or taking an antipsychotic without evidence of schizophrenia; ●One factor was associated with an increased risk of potential underuse – activities of daily living dependencies  ●Moderate to severe pain and those taking an anxiolytic/hypnotic were at significantly increased risk of inappropriate use (rather than appropriate use) |
| J. T. Hanlon et al. (2015)* | Quantitative – medical/pharmacy records | Overuse/underuse  Inappropriate use (non-adherence) | **-** | ●Mild-moderate dementia: ethnicity and polypharmacy and mental health illness significantly more likely to underuse only or both under and inappropriate use – same result for antidepressant users  ●Severe dementia: co-morbidities increased overuse and inappropriate use risk  ●Antipsychotic use associated with all three types (overuse, inappropriate use, or both) |
| J. Raynsford et al. (2020) | Quantitative – medical records | Monitoring  Drug errors – wrong dose, unclear directions | ●Lack of clarity whether tests should be done by primary or secondary care  ●50% of errors due to poor communication from secondary care  ●50% due to instructions from  secondary care being missed by GPs  ●Poor understanding of shared care guidelines | ●Patient not attending despite requests from surgery  ●Patient being abroad for an extended period |

| **Author & Year** | **Data type & Collection method** | **Safety challenge(s) with aetiological data** | **Aetiology of safety challenge(s)** | |
| --- | --- | --- | --- | --- |
|  |  |  | **Prescriber-related** | **Patient-related** |
| C. Parsons et al. (2012)* | Quantitative – medication administration records | PIM | ●Correlation observed between number of medications prescribed and occurrence of PIM, using Spearmans Correlation test (*p*<0.001) | **-** |
| W. Y. Khawagi et al. (2021)* | Quantitative – Clinical database | PHP Monitoring | ●Practices in the most deprived localities – patients had higher odds of PHP compared to patients from least deprived localities (adjusted OR 1.10, 95% CI 1.03 to 1.17)  ●Practices from more deprived localities (quintiles 3-5) – patients had higher odds of inadequate monitoring compared to patients from least deprived localities | ●PHP was associated with >10 repeat prescriptions (adjusted OR 30.22)  ●PHP risk increased with age until 35-44 years old where it started decreasing (adjusted OR 2.34, 95% CI 2.26 to 2.42)  ●Females were at more risk of PHP than men (adjusted OR 1.43, 95% CI 1.41 to 1.45)  ●Females were at more risk of inadequate monitoring than men (adjusted OR 1.12, 95% CI 1.05 to 1.20)  ●Patients with >10 prescriptions had a lower risk of inadequate medication monitoring than patients with 0–1 repeat prescriptions (adjusted OR 0.35, 95% CI 0.29 to 0.41)  ●Patients aged >74 had a lower risk than patients aged <25 (adjusted OR 0.40, 95% CI 0.31 to 0.51) |
| K. Voigt et al. (2016) | Mixed – interviews & patient medical records | PIM | ●Lack of communication regarding prescriptions of medications and referral of clinical information  ●Uncritical prescribing by psychiatrists  without a holistic approach | **-** |
| A. Hiance-Delahaye et al. (2018)* | Quantitative - interview | PIP | **-** | ●Polypharmacy was associated with PIP of ADs with adjusted OR 5-9 drugs 2.61 (95% CI 1.11– 6.16) and OR ≥10 drugs 2.69 (95% CI 1.06–6.87)  ●Longer duration of symptoms, the higher the risk of PIP of ADs, with an adjusted OR 2.82 (95% CI 1.42–6.99) for people suffering from depressive and/or anxiety symptoms for ≥28 years relative to those who reported symptoms for ≤7 years |

*Data derived from risk factor correlations; ADE = Adverse drug event; DDI = Drug-Drug interaction; AD = Antidepressant; PRN = when required; ADHD = Attention deficit hyperactivity disorder; PHP = Potentially Hazardous Prescribing; PIM = Potentially inappropriate medication; PIP = Potentially inappropriate prescribing
