# Supplementary material for: Understanding the medication safety challenges for patients with mental illness in primary care: a scoping review
Source: BMC Psychiatry. 2023 Jun 12;23:417. doi: 10.1186/s12888-023-04850-5 (PMC10258931; doi:10.1186/s12888-023-04850-5)
Supplement: Supplementary file 7 — Supplementary Material 7 - Data summary of 18 intervention studies [file 12888_2023_4850_MOESM7_ESM.docx]

**Data summary of 18 intervention studies**

| **Author & Year** | **Country** | **Primary care setting** | **Study population** | **Intervention** |
| --- | --- | --- | --- | --- |
| J. S. Bell et al. (2006) | Australia | Pharmacy | Patients with MI | Pharmacist reviews |
| N. Gisev et al. (2010) | Australia | CMHT | Patients with MI | Pharmacist reviews |
| D. Wucherer et al. (2017) | Germany | GP practice | Dementia patients | Pharmacist computer based home medication assessments |
| M. Stuhec et al. (2019) | Slovenia | Nursing home | Patients with MI | Pharmacist reviews |
| M. Stuhec et al. (2021) | Slovenia | GP practice | Patients with MI | Pharmacist reviews |
| M. Rubio-Valera et al. (2013) | Spain | Pharmacy | Patients with MI | Community pharmacist educational intervention for patients |
| C. F. Johnson et al. (2020) | UK | CMHT | Patients with MI | 3 part quality improvement intervention: ●individualised prescriber patient-level feedback summaries after each reconciliation cycle ●run charts demonstrating the proportion of patients with ≥1 psychotropic medicine discrepancy ●a planned face-to-face meeting with each CMHT to discuss and reflect on progress |
| J. Raynsford et al. (2020) | UK | GP practice | Patients with MI | Specialist mental health pharmacy team medicines optimisation within GP practice |
| J. C. Fortney et al. (2011) | USA | GP practice | Patients with MI | Telemedicine-based collaborative care from 5 types of provider |
| J. A. Sirey et al. (2017) | USA | GP practice* | Patients with MI | Treatment Initiation and Participation Program - identify barriers and develop personal adherence strategies |
| S. Priebe et al. (2013) | UK | CMHT | Patients with MI | Patient financial incentives for each depot injection over a 12 month period |
| S. Priebe et al. (2016) | UK | CMHT | Patients with MI | Discontinued £15 financial incentive - 2 year follow-up post intervention |
| S. Bhat et al. (2018) | USA | GP practice* | Patients with MI | Pharmacist-led multidisciplinary telemonitoring service |
| L. Dou et al. (2020) | China | Community-dwelling | Patients with MI | 686 project - free essential drugs and follow-up |
| L. Hoffman et al. (2003) | USA | Pharmacy | Patients with MI | Educational intervention for patients and prescribers  1. Health Plan Employer Data and Information Set 2. Information regarding the importance of medication adherence |
| M. E. Corden et al. (2016) | USA | GP practice* | Patients with MI | Digital intervention (MedLink) - mobile app that provided dose reminders, information and surveys of symptoms and side effects |
| O. H. Brook et al. (2005) | Netherlands | Pharmacy | Patients with MI | Community pharmacy-based coaching program |
| D. Velligan et al. (2013) | USA | CMHC | Patients with MI | 3 treatment groups  1. PharmCAT  2. Med-eMonitor  3. Treatment as usual |

*setting converted to UK equivalent; CMHC = Community mental health clinic; CMHT = Community mental health team; GP = General practitioner; MI = Mental illness; UK = United Kingdom; USA = United States of America
