# Supplementary material for: Understanding the medication safety challenges for patients with mental illness in primary care: a scoping review
Source: BMC Psychiatry. 2023 Jun 12;23:417. doi: 10.1186/s12888-023-04850-5 (PMC10258931; doi:10.1186/s12888-023-04850-5)
Supplement: Supplementary file 8 — Supplementary Material 8 - Intervention studies and reported outcomes full dataset [file 12888_2023_4850_MOESM8_ESM.docx]

Intervention studies and reported outcomes full dataset

| Author & Year | Country | Primary care setting | Study population | Aim | Intervention | Outcomes |
| --- | --- | --- | --- | --- | --- | --- |
| J. S. Bell et al. (2006) | Australia | Pharmacy | MH patients | Classify ADEs & make drug, prescriber, and patient recommendations | Pharmacist home medicines reviews | ●274/304 recommendations accepted (90%) ●25/304 recommendations considered (8%) ●5/304 recommendations rejected (2%) ●Most common findings: suspected and potential ADRs, potential interactions, and patients taking additional medication unbeknown to their GP ●Most frequent recommendations: switch drug, suggest non-drug treatment, start new drug, and changing dosage schedule |
| N. Gisev et al. (2010) | Australia | CMHT | MH patients | Evaluate comprehensive medicine reviews for CMHTs | Pharmacist-led medication reviews | ●81% recommendations appropriate  ●69% recommendations were likely to be implemented ●77% reviews deemed to potentially have a positive clinical impact  ●Less than 11% of recommendations were considered unlikely to be implemented. The W score of 0.40 (P<0.001) indicated moderate agreement |
| D. Wucherer et al. (2017) | Germany | GP practice | Dementia patients | Evaluate collaborative dementia care management | Pharmacist computer based home medication assessments | ●92.8% had at least one DRP detected ●64.1% had 1-3 DRPs detected ●27.3% had 4-7 DRPs detected ●1.4% had 8-12 DRPs detected |

| Author & Year | Country | Primary care setting | Study population | Aim | Intervention | Outcomes |
| --- | --- | --- | --- | --- | --- | --- |
| M. Stuhec et al. (2019) | Slovenia | Nursing home | MH patients | Evaluate impact of clinical pharmacist reviews on quality of life & quality of pharmacotherapy | Clinical psychiatric pharmacist medication reviews | ●Mean number of medications per patient decreased from 12.2 to 10.3 (*p*<0.05) ●Type X pDDI decreased by 33.3% (*p* = 0.004) ●Type D pDDI decreased by 42.6% (*p* = 0.004) ●PIMs decreased from 10.6% to 8.4% (*p* = 0.025) ●% of recommendations accepted 29.2% ●Increase in QoL measure for 70.8% of patients (*p*<0.05) ●Largest reduction in anxiety & depression - decreasing from 75% to 58.3% |
| M. Stuhec et al. (2021) | Slovenia | GP practice | MH patients | Evaluate  whether CP interventions have a long-term impact on the quality of medication prescribing in  geropsychiatric patients | Clinical psychiatric pharmacist medication reviews | ●Number of medications decreased by 9.5% (*p*<0.05) ●Recommendations accepted = 55% ●75% of patients had all proposed interventions accepted ●PIMs decreased by 49% (*p*<0.05) ●Number of pXDDIs decreased from 8 to 1 (*p*<0.05) |
| M. Rubio-Valera et al. (2013) | Spain | Pharmacy | MH patients | Evaluate the impact of a clinical pharmacist intervention on primary care patients who have started antidepressant medication | Community pharmacist educational intervention for patients | Adherence to antidepressants ●3 month follow-up - CPI 83.3% compared to control 67.7% ●6 month follow-up - CPI 67.3% compared to control 46.3%  ●Statistically significant improvement in HRQOL *p* = 0.038 ●No statistically significant difference in symptom severity (main analysis *p* = 0.432) (PP analysis *p* = 0.297) or satisfaction with the pharmacy (main analysis *p* = 0.20) (PP analysis *p* = 0.270) |

| Author & Year | Country | Primary care setting | Study population | Aim | Intervention | Outcomes |
| --- | --- | --- | --- | --- | --- | --- |
| C. F. Johnson et al. (2020) | UK | CMHT | MH patients | Increase the proportion of patients with no psychotropic drug  discrepancies at the CMHT–general practice interface | 3 part quality improvement intervention: ●individualised prescriber patient-level feedback summaries after each reconciliation cycle ●run charts demonstrating the proportion of patients with ≥1 psychotropic medicine discrepancy ●a planned face-to-face meeting with each CMHT to discuss and reflect on progress | CMHT-1 achieved a continuous non-statistically significant improvement in medicines reconciliation accuracy and a reduction in discrepancy rate per patient (χ2 = 13.05, d.f. = 3, *p* = 0.004, Cramer’s V = 0.2198). CMHT 2 & 3 did not achieve that. |

| Author & Year | Country | Primary care setting | Study population | Aim | Intervention | Outcomes |
| --- | --- | --- | --- | --- | --- | --- |

| J. Raynsford et al. (2020) | UK | GP practice | MH patients | Investigate the  contribution a specialist mental health clinical pharmacy  team could make to medicines optimisation for patients  on the SMI register in primary care | Specialist mental health pharmacy team medicines optimisation within GP practice | % of interventions graded 1-4 1. Very significant hospital admission prevented 5.8% 2. Significant improved outcome if changed 40.4% 3. Somewhat significant understanding increased 30.8% 4. No clinical significance 23.1%  Scales used adapted from Nathan et al. ^(127)^ |
| --- | --- | --- | --- | --- | --- | --- |
| J. C. Fortney et al. (2011) | USA | GP practice | MH patients | Test whether collaborative care improves antidepressant adherence | Telemedicine-based collaborative care from 5 types of provider | Self-reported adherence significantly improved ●6 months OR = 2.11; 95% CI, 1.0-4.4; *p* = 0.04 ●12 months OR = 2.72; 95% CI, 1.4-5.4; *p*<0.01 |
| J. A. Sirey et al. (2017) | USA | GP practice* | MH patients | Evaluate the effectiveness of a psychosocial intervention to improve early  adherence among older patients who are newly initiated on an antidepressant | Treatment Initiation and Participation Program (TIP) - identify barriers and develop personal adherence strategies | TIP group ●6 weeks = 5 times more likely to be adherent (OR = 5.54; 95% CI, 2.57-11.96; χ2 = 19.05; *p*<0.001) ●6 & 12 weeks = 3 times more likely to be adherent (OR = 3.27; 95% CI, 1.73-6.17; χ2 = 13.34; *p*<0.001)  ●Significant early improvement (24.9% change from baseline) in depressive symptoms at 6 weeks (95% CI, 13.9-35.9; t337 = 4.46; adjusted *p*<0.001) |

| Author & Year | Country | Primary care setting | Study population | Aim | Intervention | Outcomes |
| --- | --- | --- | --- | --- | --- | --- |

| S. Priebe et al. (2013) | UK | CMHT | MH patients | Evaluate if financial incentives is effective in improving adherence to maintenance treatment with antipsychotics in patients with psychotic disorders | Patient offered £15 for each depot injection over a 12 month period | ●Baseline adherence - Control = 67%, Intervention = 69%  ●12 month adherence - Control = 71%, Intervention = 85%  ●Adjusted effect = 11.5% (95% confidence interval 3.9% to 19.0%, *p* = 0.003)  ●Adherence ≥95% achieved in 28% of the intervention group and 5% of the control group (OR = 8.21, 95% confidence interval 2.00 to 33.67, *p* = 0.003).  ●Patients in the intervention group had more favourable QoL ratings (β=0.71, 95% CI, 0.26 to 1.15, *p* = 0.002) |
| --- | --- | --- | --- | --- | --- | --- |
| S. Priebe et al. (2016) | UK | CMHT | MH patients | Evaluate whether the positive effect continues once the incentives stop | Discontinued £15 financial incentive - 2 year follow-up post intervention | ●No statistically significant difference in mean adherence between control and intervention groups ●Control - 6 months = 78%, 18 months = 74% ●Intervention - 6 months = 71%, 18 months = 68%  ●6 months −7.4%, 95% CI, 17.0 to 2.1, *p* = 0.175  ●18 months −5.7, 95% CI, 13.1%-1.7%, *p* = 0.130  ●95% adherence during the 6-month and further 18-month follow-up was not significantly different  ●6 months = OR = 0.42, 95% CI, 0.11-1.61, *p* = 0.205  ●24 months = OR = 0.42, 95% CI, 0.06-3.02, *p* = 0.392 |
| S. Bhat et al. (2018) | USA | GP practice* | MH patients | Evaluate a service to monitor  patients after antidepressant initiation or titration in primary care settings | Pharmacist-led multidisciplinary telemonitoring service | ●Identified medication nonadherence in 19%, suicidal ideation in 4%, and provided interventions in 42% of calls  ●109 interventions for 102/258 (40%) unique patients  ●Improved medication possession ratio (0.81  vs. 0.66; *p* = 0.0001)  ●Medication switch rates (Pharmacist 24% vs. PCP 5%; *p* = 0.0001) |

| Author & Year | Country | Primary care setting | Study population | Aim | Intervention | Outcomes |
| --- | --- | --- | --- | --- | --- | --- |
| L. Dou et al. (2020) | China | Community-dwelling | MH patients | Evaluate the effectiveness of the 686 on patient medication adherence | 686 project - free essential drugs and follow-up | ●686 project group = 92.6% adherence ●Non-policy group = 61.2% adherence |
| L. Hoffman et al. (2003) | USA | Pharmacy | MH patients | Evaluate the impact of mail-based physician  and educational interventions on patient adherence to  antidepressant medications | Educational intervention for patients and prescribers  1. Health Plan Employer Data and Information Set (HEDIS) 2. Information regarding the importance of medication adherence | Adherence rates ●First observation - Control = 57.4%, Intervention = 58.9%  Medication possession ratio ●Second observation - Control = 65.5%, Intervention = 66.9%  ●Third observation - Control = 50.2%, Intervention = 52.3%  HEDIS ●Second observation - Control = 56.6%, Intervention = 59.6%  ●Third observation - Control = 29.4%, Intervention = 31.4%  ●Intervention group demonstrated greater adherence compared with the control group at 90 and 180 days (*p*<0.05)  ●Intervention variable stood alone in its significant impact on adherence (*p*<0.01; CI, 1.003-1.197) |

| Author & Year | Country | Primary care setting | Study population | Aim | Intervention | Outcomes |
| --- | --- | --- | --- | --- | --- | --- |
| M. E. Corden et al. (2016) | USA | GP practice* | MH patients | Evaluate the feasibility of a digital intervention to improve antidepressant adherence | Digital intervention (MedLink) - mobile app that provided dose reminders, information and surveys of symptoms and side effects | ●Mean medication adherence = 82%  ●Adherence was 88.5% during the first four weeks and dropped to 73.0% in the last four weeks (*p* = 0.16)  ●91% continued medication at the end of the trial ●Significant decreases in depressive symptoms (PHQ-9) (*p* = 0.0005) and QIDS (*p* = 0.0008) |
| O. H. Brook et al. (2005) | Netherlands | Pharmacy | MH patients | Evaluate the effects on adherence and depressive  symptoms of a community  pharmacy-based coaching program | Community pharmacy-based coaching program | ●Intention-to-treat analysis Mean adherence - Control = 73%, Intervention = 76%  ●Per-protocol Control = 73%, Intervention = 90%  ●Only the per-protocol analysis indicated significantly better adherence in the intervention group  than in the control group *p*<0.05 |
| D. Velligan et al. (2013) | USA | CMHC | MH patients | Evaluate the effectiveness of two interventions on adherence | 3 treatment groups  1. PharmCAT 2. Med-eMonitor 3. Treatment as usual (TAU) | ●Mean adherence 1. PharmCAT = 90% 2. Med-eMonitor = 91% 3. TAU = 72% ●Adherence significantly better in the 2 active interventions (both *p*<0.0001) |

*setting converted to UK equivalent; ADR = Adverse drug reaction; APS = Antipsychotic; CMHT = Community mental health team; CPI = Community pharmacist intervention; DDI = Drug-Drug interaction; DRP = Drug-related problem; GP = General practitioner; MH = Mental health; pDDI = Potentially drug-drug interactions; PIM = Potentially inappropriate medication; pXDDI = Potential type X drug-drug interactions; QoL = Quality of life; SSRI = Selective serotonin reuptake inhibitors; Type D pDDI = Minor interactions to be avoided if possible; Type X pDDI = Major interactions which should be avoided
